# Antioxidant, antihypertensive, anti-hyperglycemic, and antimicrobial activity of aqueous extracts from twelve native plants of the Yucatan coast

**DOI:** 10.1371/journal.pone.0213493

**Published:** 2019-03-27

**Authors:** Cecilia Mónica Rodríguez-García, Jorge Carlos Ruiz-Ruiz, Leticia Peraza-Echeverría, Sergio Rubén Peraza-Sánchez, Luis Wiliunfo Torres-Tapia, Daisy Pérez-Brito, Raúl Tapia-Tussell, Francisco Gilberto Herrera-Chalé, Maira Rubí Segura-Campos, Andrés Quijano-Ramayo, Jesús Manuel Ramón-Sierra, Elizabeth Ortiz-Vázquez

**Affiliations:** 1 Unidad de Biotecnología, Centro de Investigación Científica de Yucatán A.C., Mérida, Yucatán, México; 2 Departamento de Ingeniería Química-Bioquímica, Instituto Tecnológico de Mérida, Mérida, Yucatán, México; 3 GEMBIO, Centro de Investigación Científica de Yucatán A.C., Mérida, Yucatán, México; 4 Facultad de Ingeniería Química, Universidad Autónoma de Yucatán, Mérida, Yucatán, México; 5 División de Estudios de Postgrado e Investigación, Instituto Tecnológico de Mérida, Mérida, Yucatán, México; Tallinn University of Technology, ESTONIA

## Abstract

Looking for a biotechnical potential, aqueous extracts of leaves of 12 native species used in the Mayan traditional medicine of the coastal dune and mangrove of Yucatan (Mexico) were selected to evaluate their biological activities. *Rhizophora mangle* and *Manilkara zapota* showed the highest free radical scavenging activity (3.94 ± 0.19 and 6.42 ± 0.32 μg/mL, respectively), and the highest antihypertensive activity was obtained from *Solanum donianum* (0.38 μg/mL). The anti-hyperglycemic activity of these species was also tested; the highest activities were registered with *R*. *mangle*. The antimicrobial activity of *Malvaviscus arboreus*, *S*. *donianum*, *M*. *zapota*, and *R*. *mangle* at 10% (w/v) was positive against six human pathogenic bacteria and *Bonellia macrocarpa* against one pathogenic fungus. *Solanum donianum*, *M*. *zapota*, *B*. *macrocarpa*, and *R*. *mangle* were positive against two pathogenic plant fungi. These results show that the aqueous extracts of five native plants of the Yucatan coast have potential as antioxidants, ACE inhibitors, α-amylase and α-glucosidase inhibitors, and as antimicrobials, which make their exploration for utilization in the agricultural and pharmaceutical industries a possibility.

## Introduction

Natural products coming from plants have been an axis of the Mayan traditional medicine, and they remain regularly in use today. Recently, the ethnomedicinal knowledge of 100 species from 680 registered in the Yucatan peninsula was published [1]. Nevertheless, until today, the ethnomedicinal knowledge of the coastal dune and mangrove of Yucatan State, where plant species are growing with extreme climatological conditions, has not been reported. As a consequence, they produce metabolites that could have some biological activity (e.g. antioxidant, antimicrobial), which could be a biotechnological potential target in the health and agricultural sectors.

In the last two decades, natural products have been the aim of many investigations, and the direct use of these products has been encouraged in the pharmaceutical and agricultural industries [2, 3]. For example, antioxidant compounds through their scavenging power are useful for the management of many disorders (e.g. neurodegenerative diseases, cancer, AIDS). On the other hand, to manage hypertension and reduce the risk of cardiovascular events, the major therapeutic strategy currently used is the pharmacological inhibition of the Renin Angiotensin Aldosterone System. However, angiotensin-converting enzyme (ACE)-inhibitors exhibit adverse side effects in the short and long term, including cough, disturbances in taste, and skin rashes [4]. In consequence, the plant polyphenolic compounds and proteins have been evaluated as potential natural ACE inhibitors [5]. Another metabolic disease is diabetes mellitus characterized by hyperglycemia [6]. Although there are various medications available for diabetes mellitus treatment (e.g. biguanides, sulphonylureas, and thiozolidinediones), they have exhibited a number of undesired side effects associated with their uses, so other effective pharmacological alternatives as natural products have been suggested [7].

Other human disorders are caused by bacterial and fungal pathogens, with the resistance of human pathogenic strains to antibiotics or fungicides being a particular problem [8, 9]. In the same way, fungal pathogens of crops have acquired resistance to synthetic fungicides [10].

In the present work, looking for a biotechnological potential, the biological activities of aqueous extracts of leaves of 12 native species used in the Mayan traditional medicine from the coastal dune and mangrove of Yucatan State were evaluated.

## Materials and methods

### Information of study area

Data collection were achieved through semi-structured, open-recorded, anonymous interviews with ‘key informants’ in 11 coastal villages of Yucatan State, Mexico (i.e. Celestún, Sisal, Chuburná Puerto, Chelem, Telchac Puerto, San Crisanto, Santa Clara, Dzilam de Bravo, Las Coloradas, Río Lagartos, and El Cuyo). The ‘key informants’, recognized for their knowledge about medicinal plants, were identified by talking with local people as Martin [11] suggests. The interviews were anonymous to protect the identity of the informants; only general data, such as age, gender, and mother tongue, were obtained. This study was approved by the Research Ethics Committee of the Universidad Autónoma de Yucatán, Mexico (S1 Appendix). The floristic composition of the study area is characteristic of coastal dune vegetation [12, 13, 14] and mangrove [15]. The average annual rainfall was 524 mm, with an average annual temperature of 26.5 °C [16]. The soil types of coastal dune and mangrove are arenosol, gleysol, solonchack, regosol, and histosol [17].

### Plant material

For this study we selected 12 species because they are wild and native plants from the Yucatan coast. They are used in Mayan traditional medicine, and they were available during this study, and considering our conservationist philosophy about native plants from this region, it was decided to work only with their leaves, which were collected (S1 Fig) during the rainy season of 2014. The identity of the plants was confirmed depositing specimens at "U najil tikin xiw" herbarium of the Centro de Investigación Científica de Yucatán (CICY).

### Preparation of aqueous extracts

Fresh leaves were collected from each species, and the 12 aqueous extracts were obtained using a previously published technique [18] https://dx.doi.org/10.17504/protocols.io.szwef7e. The final volume obtained ranges between 60 and 80 mL. Each aqueous extract (AE) was stored at 4 °C until use.

### Determination of phenolic compounds and flavonoid content

Phenolic compounds were determined using the Folin-Ciocalteu reagent (Sigma-Aldrich, St. Louis, MO, USA) according to Georgé et al. [19] https://dx.doi.org/10.17504/protocols.io.sfkebkw. The flavonoid content was determined using the aluminum chloride (Fermont, Monterrey, NL, Mexico) method [20] https://dx.doi.org/10.17504/protocols.io.sfnebme. Catechin (Sigma-Aldrich) was used as the standard and the results were expressed as μg/mL of catechin equivalents (CE).

### Free radical scavenging activity

The method of Meda et al. [21], with some modifications https://dx.doi.org/10.17504/protocols.io.sfpebmn, was followed to measure the free radical scavenging assay using 1,1-diphenyl-2-picrylhydrazyl (DPPH, Sigma-Aldrich). The control was distilled water and the standard the ascorbic acid. The free radical scavenging activity, expressed as percentage of inhibition, was calculated using the formula (A0 − A1)/A0 − 100, where A0 was the control absorbance, measured at 517 nm, and A1 that of the sample. The antioxidant activity was quantified by a regression analysis of the percentage of free radical scavenging (%) versus the phenolic compound concentration in the aqueous extract; this was defined as an IC_50_ value, which is the amount of antioxidant necessary to decrease the initial DPPH radical concentration by 50%.

### Angiotensin-converting enzyme inhibitory assay

Inhibitory activity of aqueous extracts was analyzed following a method by Hayakari et al. [22] https://dx.doi.org/10.17504/protocols.io.tb4eiqw. Hippuryl-L-histidyl-L-leucine (HHL) (Sigma) was hydrolyzed by ACE to yield hippuric acid and histidyl-leucine. This method relies on the colorimetric reaction of hippuric acid with 2, 4, 6-trichloro-s-triazine (TT) (Sigma). The tests were performed in triplicate and antihypertensive activity was quantified by a regression analysis of ACE inhibitory activity (%) versus phenolic compounds concentration in aqueous extract and defined as an IC_50_ value, that is, the concentration of phenolic compounds required to produce 50% ACE inhibition under the conditions described. For angiotensin-converting enzyme inhibitory assay, lisinopril (antihypertensive drug) was used as control.

### Anti-hyperglycemic activity

#### *In vitro* α-amylase inhibitory assay

The assay was carried out following the protocol reported by Dineshkumar et al. [7] https://dx.doi.org/10.17504/protocols.io.tgfejtn. The assay was performed in triplicate. The α-amylase inhibitory activity was calculated using the formula (Ac^+^) − (Ac^−^) − (As − Ab)/(Ac^+^) − (Ac^−^) × 100, where Ac^+^, Ac^−^, As, Ab are defined as the absorbance, at 595 nm of 100% enzyme activity (only solvent with enzyme), 0% enzyme activity (only solvent without enzyme), the test sample (with enzyme), and a blank (a test sample without enzyme), respectively.

#### *In vitro* α-glucosidase inhibitory assay

The *in vitro* α-glucosidase inhibitory assay was performed according to Dineshkumar et al. [7] https://dx.doi.org/10.17504/protocols.io.tgfejtn. The α-glucosidase inhibitory activity was calculated using the formula (Ac^+^)–(Ac^−^)–(As − Ab)/(Ac^+^) − (Ac^−^) × 100, where Ac^+^, Ac^−^, As, and Ab are defined as the absorbance at 405 nm, of 100% enzyme activity (only solvent with enzyme), 0% enzyme activity (only solvent without enzyme), test sample (with enzyme), and a blank (a test sample without enzyme), respectively. Reagents to evaluate anti-hyperglycemic activity were of analytical grade and purchased from Sigma-Aldrich. For anti-hyperglycemic activity, acarbose (anti-hyperglycemic drug) was used as control.

### Antimicrobial activity

#### Human pathogenic microbial material

Several human pathogenic strains were employed to test the antimicrobial activity of plant extracts: *Escherichia coli* ATCC 25922, *Escherichia coli* O157:H7, *Vibrio cholerae* ATCC 14035, *Pseudomonas aeruginosa* ATCC 27853, *Listeria monocytogenes* ATCC 15313, and *Staphylococcus aureus* ATCC 25923. The fungus employed in this work was *Candida albicans* ATCC 1023. All bacterial strains were grown in Luria Broth at 37 °C and stored in 20% glycerol at −20 °C. *Candida albicans* was grown in Sabouraud agar at 37 °C.

#### Phytopathogenic fungal material

Five phytopathogenic fungi were isolated from papaya (*Carica papaya* L.), physic nut (*Jatropha curcas* L.), vanilla (*Vanilla planifolia* Jacks. ex Andrews), and banana (*Musa acuminata* C.) crops in different states of southeast Mexico (S1 Table). Fungal isolates were obtained from lesions on leaves, flowers, and fruits of infected plants https://dx.doi.org/10.17504/protocols.io.sgsebwe. Spore suspension was prepared in sterile distilled water by harvesting asexual reproductive structures from 7-day old cultures of each isolate, and the concentration was adjusted to 1 × 10^6^ spores/mL.

The total genomic DNA of mycelium from each fungus was extracted using the method described by Tapia-Tussell et al. [23] https://dx.doi.org/10.17504/protocols.io.shteb6n and diluted to a final concentration of 20 ng/μL. PCR products (i.e. 500 and 800 base pairs) were purified and sequenced by Macrogen Inc. in Korea. Sequences were edited and assembled using the BioEdit Sequence Alignment program [24]. Blast searches were performed against the NCBI nucleotide databases.

*Mycosphaerella fijiensis* strain C1233 (accession number IMI 392976, International Mycological Institute, CABI Bioscience Centre Egham, UK), isolated from banana of Yucatan state was previously identified using PCR, and the asexual spores were obtained according to Peraza-Echeverría et al. [25] https://dx.doi.org/10.17504/protocols.io.sgmebu6.

#### Assessment of human antimicrobial activity

https://dx.doi.org/10.17504/protocols.io.tg9ejz6 To prepare the inoculum, individual colonies of human pathogens were taken from a previous overnight culture and dissolved in saline solution (0.85% NaCl) to reach a concentration of 0.5 McFarland (1 × 10^8^ CFU/mL). After inoculation, the final concentration of bacteria in the medium was 5 × 10^6^ CFU/mL. The inoculum was spread over the plates. A sterile paper disk was placed on the plates. Different amounts of the plant extracts (0.2 to 2%) were dispensed on the paper disk. Sterile water and ampicillin (50 μg) were used as controls. The plates were incubated at 37 °C for 24 h. A transparent ring around the paper disk expressed antibacterial activity [26].

The minimum inhibitory concentration (MIC) was determined by micro-dilution assay [26], which employs different concentrations of extract in the range of 1 to 3.4%. The final volume of 500 μL was used, with a concentration of inoculum of 5 × 10^6^ CFU/mL of human pathogen. Since extracts that present turbidity could cause interference, each dilution was performed separately. The inoculated medium was incubated at 37 °C for 12 h and the optical density (OD) at 600 nm was measured to determine the growth of bacteria. The assay was repeated five times.

#### Detection of anti-phytopathogenic fungal activity

*In vitro* antifungal susceptibility by the broth dilution method was used to find the MIC of each aqueous extract against six phytopathogenic fungi. Four concentrations of each extract were assayed: 1, 2.5, 5, and 10% (v/v) in PDB (potato dextrose broth, Difco) https://dx.doi.org/10.17504/protocols.io.wyqffvw. Each one was inoculated with the corresponding fungus at 20 spores/μl (final concentration), and incubated for three days in the dark at 26‒28 °C. PDB medium was used as a negative control. A commercial fungicide (miconazole nitrate, 20 mg/mL) was added to the positive control at a final concentration of 0.2 ng/μL. After three days of incubation, 25 μL was taken from each concentration tested and poured into Petri dishes (35 mm × 10 mm, polystyrene dish, Corning) with PDA (potato dextrose agar, Difco) and incubated at 26 ± 2 °C at 12/12 h photoperiod for 7 days (two replicates per concentration). Evaluations were made on day 7. The MIC was determined as the lowest concentration of aqueous extract preventing the growth of macroscopically visible mycelium.

### Chromatographic profile

The HPLC-DAD equipment used was an Agilent Series 1290 infinity with vacuum degasser, quaternary pump, autosampler, thermostated column compartment and photodiode array detector (DAD). Data analysis was performed with Agilent HPLC EZChrom software. The chromatographic separation was performed at 35 °C on a Grace Alltima C18 column (4.6 mm × 250 mm, 5 μm) with a flow rate of 1 mL/min, and the injection volume was 20 μL. Mobile phase A consisted of ultrapure type 1 water (Simplicity Water Purification System, Millipore) adjusted to pH 2.5 with trifluoroacetic acid (TFA), and mobile phase B contained acetonitrile (ACN). The gradient was programmed as follows: 0–3 min, 5% B; 3–43 min, 5–30% B; 43–73 min, 30–85% B; 73–75 min, 85–5% B. Simultaneous monitoring was performed for determination at 254 and 350 nm. ACN and methanol (MeOH) were HPLC grade (JT Baker) and TFA HPLC grade (Sigma-Aldrich). Prior to injection, 10% MeOH was added to each sample to improve solubility https://dx.doi.org/10.17504/protocols.io.wy9ffz6

### Statistical analysis

All experiments were performed, at least, twice with three replicates. One way ANOVA was run to determine free radical scavenging activity and anti-hyperglycemic activity. The least significant difference (LSD) multiple-range test was used to establish differences among extracts. All analyses were processed with the Statgraphics Plus version 5.1 software. MS Excel spreadsheets were also utilized to summarize the data using descriptive statistics (percentages, averages, standard errors) and to draw bar charts.

## Results and discussion

### Medicinal plants from the Yucatan coast

A total of 12 native species (Table 1) grown naturally on the coastal dune and mangrove were mentioned as medicinal plants by 21 ‘key informants’ (16 females and 5 males) whose age ranged from 25 to 97. Although around 200 native species of the coastal dune and mangrove of Yucatan have been reported [27], their medicinal use is not well-known (6%) by people of the coastal towns of Yucatan, probably due to actual access to health care clinics, in addition to young people’s indifference to learning about the medicinal properties of plants [1], which has had a deleterious effect on ethnomedicinal and botanical knowledge. The plant parts used to cure certain disorders are leaves, bark, flowers, fruits, tuber, and roots, with the leaves (43%) being the most used as also mentioned by Méndez-González et al. [1]. The most common disorders treated with native medicinal plants of the coastal dune, and mangrove of Yucatan, are diarrhoea, diabetes, high cholesterol, kidney stones, and wounds. The most commonly used species for, at least, three human disorders are *C*. *uvifera* and *M*. *zapota* (Table 1).

**Table 1:** Native medicinal plants of coastal dune and mangrove of Yucatan selected in this study.

Scientific name	Family	Mayan name^a^	Habit^b^	Voucher	PU^c^	Human disorders
(Abbreviation)						
*Anthurium schlechtendalii* Kunth *ssp*. *Schlechtendalii* (As)	Araceae	*pool boox*	H	CM Rdgz et al. 147	R	Kidney stones
*Bonellia macrocarpa* (Cav.) B. Ståhl & Källersjö *ssp*. *macrocarpa* (Bm)	Theophrastaceae	*ya’ax k’iix le’ che’*	S	CM Rdgz et al. 144	F	Threw up
*Bravaisia berlandieriana* (Nees) T.F. Daniel. (Bb)	Acanthaceae	*juluub*	SS	CM Rdgz et al. 161	L	Wounds
*Bursera simaruba* (L.) Sarg. (Bs)	Burseraceae	*chakaj*	T	CM Rdgz & A. Dorantes 181	L	Indigestion and diarrhea
*Capraria biflora* L. (Cb)	Scrophulariaceae	*boox*	SS	CM Rdgz & A. Dorantes 177	L	Diarrhea and hemorrhoids
*Coccoloba uvifera* (L.) L. (Cu)	Polygonaceae	*ni’che’*	T	CM Rdgz et al. 164	L	Sores,
					B	throat infection,
					Fr	high cholesterol
*Echites umbellatus* Jacq. (Eu)	Apocynaceae	*aak’its*	L	CM Rdgz & A. Dorantes 179	T	Wounds
*Ipomoea pes-caprae* (L.) R. Br. (Ip)	Convolvulaceae		H	CM Rdgz et al. 171	L	Kidney stones
*Malvaviscus arboreus* Cav. (Ma)	Malvaceae	*taman ch’ iich’*	S	CM Rdgz et al. 172	F	Cough
*Manilkara zapota* (Linnaeus) van Royen (Mz)	Sapotaceae	*ya’*	T	CM Rdz et al. 159	L	High cholesterol and circulation disorders,
					B	dysentery
*Rhizophora mangle* L. (Rm)	Erythroxylaceae	*ta’ab che’*	T	CM Rdgz et al. 153	B	Diabetes and vitiligo
*Solanum donianum* Walp. (Sd)	Solanaceae	*puuch’ uuk’*	H	CM Rdgz & A. Dorantes 180	Fr	Diabetes

^a^Flora Digital: Península de Yucatán, Herbario CICY, Unidad de Recursos Naturales.

http://www.cicy.mx/sitios/flora%20digital/ficha_virtual.php?especie=820 (14/jun/2016)

^b^H = herb, L = liana, S = shrub, SS = subshrub, T = tree

^c^PU = part used, B = bark, F = flower, Fr = fruit, L = leaf, R = root, T = tuber

### Antioxidant activity

The 12 aqueous extracts showed the presence of polyphenols, which was expected because all the plant species produce these secondary metabolites to protect themselves from other organisms [28]. *Anthurium schlechtendalii* showed the lowest content (226.71 μg GAE/mL of extract), while *M*. *zapota* exhibited the highest content (1,738.47 μg GAE/mL of extract). The flavonoid contents of the extracts in terms of catechin equivalent were between 117.20 μg CE/mL of extract and 2930.07 μg CE/mL of extract (Table 2). *Anthurium schlechtendalii* also showed the lowest content (226.7 μg GAE/mL) while *S*. *donianum* exhibited the highest content (2930 μg CE/ mL). This value was lower compared to the one reported for another wild plant, the *S*. *ferrugineum* leaf methanolic extract (180 mg CE/g dry tissue) [29], which could be due to dilution of metabolites in the aqueous solution.

**Table 2:** Total phenol and flavonoid content of aqueous extracts^1^.

Aqueous extracts	Total phenol content	Flavonoid content
	(μg GAE/mL)	(μg CE/mL)
*Anthurium schlechtendalii*	226.71 ± 24.13^a^	117.20 ± 4.73^a^
*Bonellia macrocarpa*	461.69 ± 17.24^e^	358.21 ± 11.05^b^
*Bravaisia berlandieriana*	242.80 ± 2.76^a^	601.45 ± 11.05^c^
*Bursera simaruba*	572.35 ± 19.30^f^	1,566.59 ± 34.71^hi^
*Capraria biflora*	309.10 ± 6.89^b^	650.54 ± 36.29^d^
*Coccoloba uvifera*	346.15 ± 9.65^c^	360.44 ± 7.89^b^
*Echites umbellata*	384.66 ± 13.10^d^	845.80 ± 22.09^f^
*Ipomoea pes-caprae*	488.99 ± 21.37^e^	1,616.80 ± 33.14^i^
*Malvaviscus arboreus*	483.63 ± 5.52^e^	761.00 ± 25.25^e^
*Manilkara zapota*	1,738.47 ± 5.52^i^	1,199.50 ±36.29^g^
*Rhizophora mangle*	1,345.54 ± 4.14^h^	1539.81 ± 3.16^h^
*Solanum donianum*	663.52 ± 17.24^g^	2,930.07 ± 15.78^j^

^1^Different superscripts, in the same column, indicate significant differences

**Fig 1:**
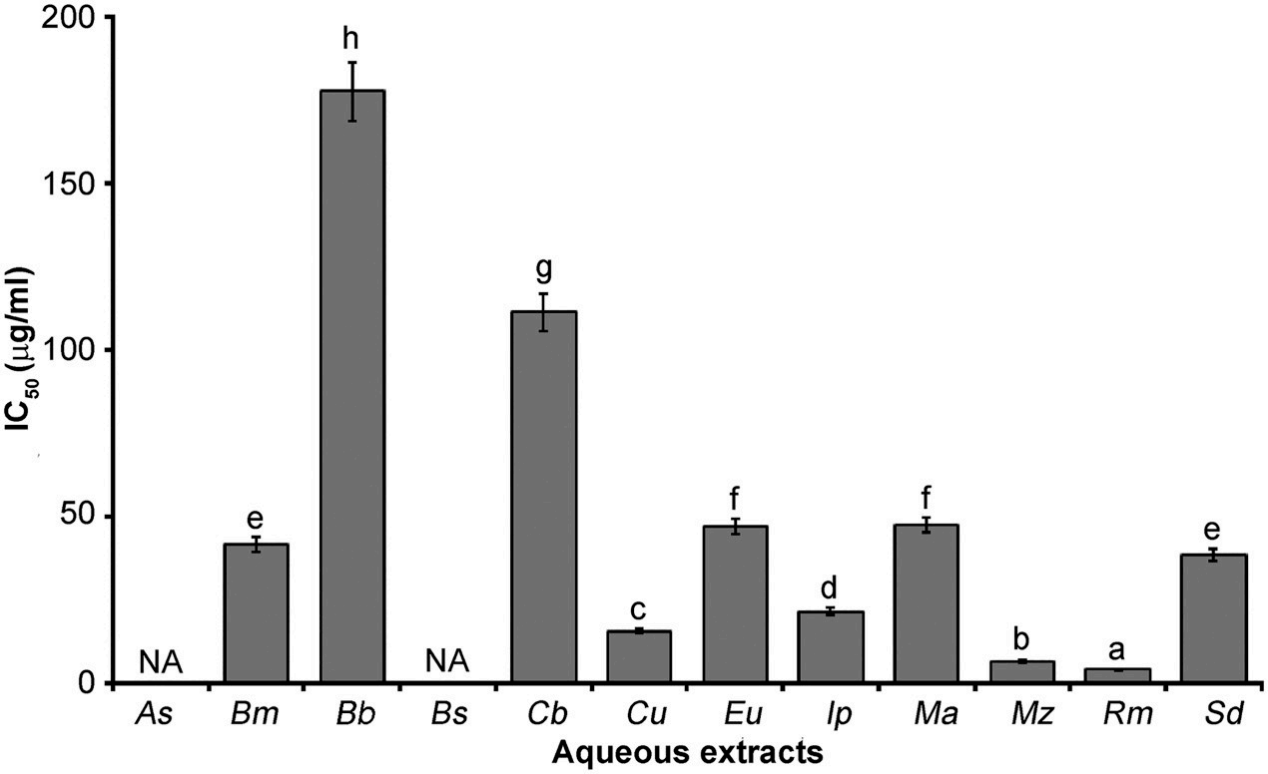
Antioxidant activity of the aqueous extracts from Mayan medicinal plants. As = *Anthurium schlechtendalii*, Bm = *Bonellia macrocarpa*, Bb = *Bravaisia berlandieriana*, Bs = *Bursera simaruba*, Cb = *Capraria biflora*, Cu = *Coccoloba uvifera*, Eu = *Echites umbellata*, Ip = *Ipomoea pes-caprae*, Ma = *Malvaviscus arboreus*, Mz = *Manilkara zapota*, Rm = *Rhizophora mangle*, Sd = *Solanum donianum*, NA = Not active, a-h = different letters in the bars indicate statistical difference (p < 0.05).

Polyphenols and flavonoids are known to have antioxidant activity, and it is likely that the activity of the aqueous extracts in this study is due to these compounds. This activity is believed to be mainly due to their redox properties, which plays an important role in adsorbing and neutralizing free radicals, quenching singlet and triplet oxygen, or decomposing peroxides [30]. Concerning the free radical scavenging activities measured, Fig 1 shows the amount of each extract needed (μg/mL of extract) for 50% inhibition (IC_50_). According to Ferry et al. [31], the lower the IC_50_, the higher the antioxidant capacity. Ascorbic acid (control) and aqueous extracts showed scavenging activity in a concentration-dependent manner. While the positive control ascorbic acid showed the lowest IC_50_ value (0.5 μg/mL), the aqueous extract values ranged between 3.94 and 177.75 μg/mL, with lower values exhibited by *Rhizophora mangle* (3.94 μg/mL) and *M*. *zapota* (6.42 μg/mL). These species are traditionally used in different countries for medical applications. Cruz et al. [32] evaluated the antioxidant properties of *R*. *mangle* from Guatemala; these authors obtained organic extracts from leaves and reported an IC_50_ = 150 μg/mL. This value is 38 times greater than that reported in the present study; the content of phenolic compounds of *R*. *mangle* that grows in Yucatan was double. In another study, Chanda and Nagani [33] reported an IC_50_ = 160 μg/mL for an aqueous extract of *M*. *zapota* from India. The value is 25 times greater than that reported in our study, where *M*. *zapota* leaves from Yucatan exhibited a content of total phenols that was 17 times higher and a content of flavonoids that was 63 times higher than that of *M*. *zapota* extract from India. Furthermore, Kaneria and Chanda [34] reported an antioxidant activity of acetone extract leaves of *M*. *zapota* from Gujarat, India, with an IC_50_ = 7.6 μg/mL, a value very close to that we found. The content and composition of polyphenols influence the antioxidant capacity of the extracts. This indicates that the anti-oxidative activities observed could be due to the synergistic effect of many compounds that may be present in these extracts. This study showed that aqueous extracts of *R*. *mangle* and *M*. *zapota* have good free radical scavenging ability and can be used as inhibitors or scavengers of free radicals, possibly acting as primary antioxidants.

### Antihypertensive activity

**Fig 2:**
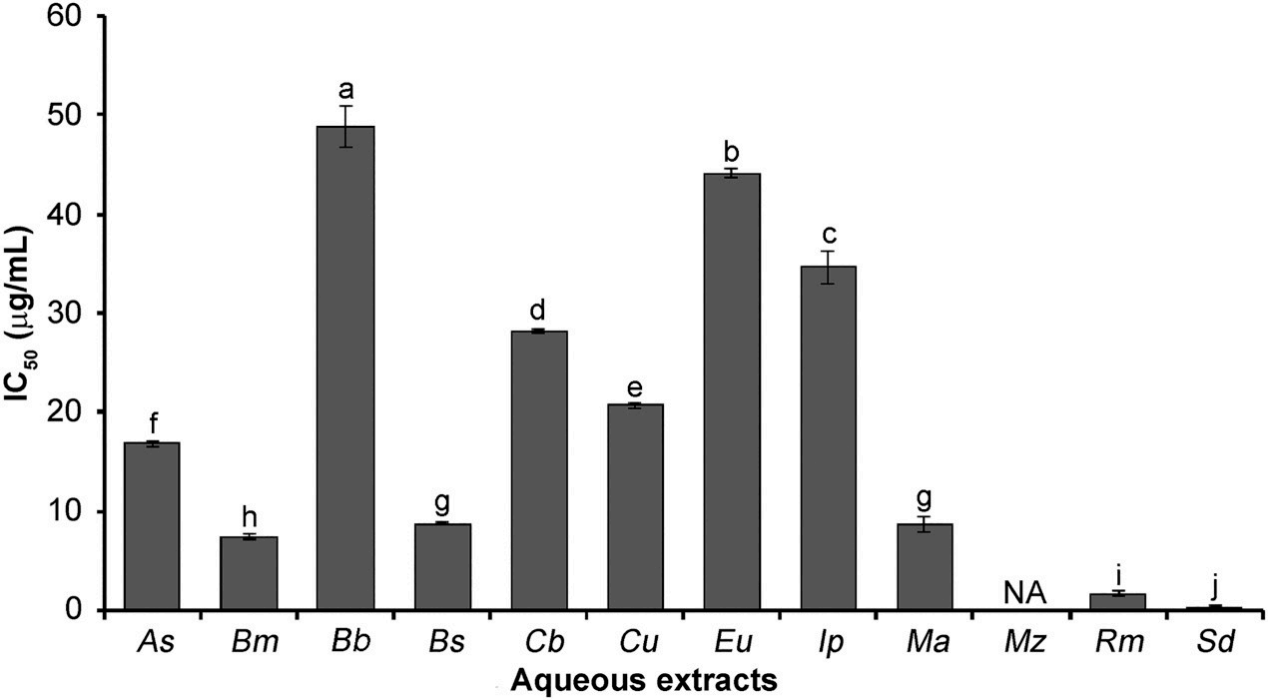
Antihypertensive activity of the aqueous extracts from Mayan medicinal plants. As = *Anthurium schlechtendalii*, Bm = *Bonellia macrocarpa*, Bb = *Bravaisia berlandieriana*, Bs = *Bursera simaruba*, Cb = *Capraria biflora*, Cu = *Coccoloba uvifera*, Eu = *Echites umbellata*, Ip = *Ipomoea pes-caprae*, Ma = *Malvaviscus arboreus*, Mz = *Manilkara zapota*, Rm = *Rhizophora mangle*, Sd = *Solanum donianum*, NA = Not active, a-j = different letters in the bars indicate statistical difference (p < 0.05).

Lisinopril (control) and aqueous extracts showed antihypertensive activity in a concentration-dependent manner, showing the positive control lisinopril the lowest IC_50_ (12.36 μg/mL). Among the aqueous extracts, *Solanum donianum* had the highest ACE inhibitory effect (IC_50_ = 0.38 μg/mL) (Fig 2). The biological potential of aqueous extracts of *S*. *donianum* and *R*. *mangle* (IC_50_’s of 0.38 and 1.75 μg/mL, respectively) is higher compared to the lyophilized aqueous extract of *Salvia hispanica* seed (IC_50_ = 5.6 μg/mL) [35]. Arenas-Carvajal et al. [36] studied the *in vitro* inhibiting effect on ACE of an extract of *Salvia scutellaroides*, demonstrating the interaction of tannins with the catalytic site of ACE, supporting the proposal that allosteric relationships affect enzyme conformation and its interaction with the substrate, these compounds being present in aqueous extracts responsible for biological activity. In this sense, vegetal extracts contain a varied distribution of tannins, phenolic compounds, glycosides, saponins, steroids, terpenoids, and anthraquinones. According to Siddesha et al. [37], the correlation between the content of phytochemicals and ACE-inhibitory activity suggests that the high content of glycosidic and phenolic compounds could be involved in exerting ACE-inhibitory activity. Flavonoids are the largest group of polyphenolic compounds found in higher plants [38], where they contribute to insect repulsion as well as protection against viral, fungal, and bacterial infections and UV light [39]. Flavonoids as ACE inhibitors for regulating blood pressure have been studied during the past decades; most of them have proved to be effective in suppressing the activity of ACE [40]. In the present study, *S*. *donianum* exhibited both the highest IC_50_ and the highest content of flavonoids (Table 2). The results of this study showed that the aqueous extract of *S*. *donianum* has good ACE inhibitory ability. This is the first report on antihypertensive activity of *S*. *donianum*.

### Anti-hyperglycemic activity

**Fig 3:**
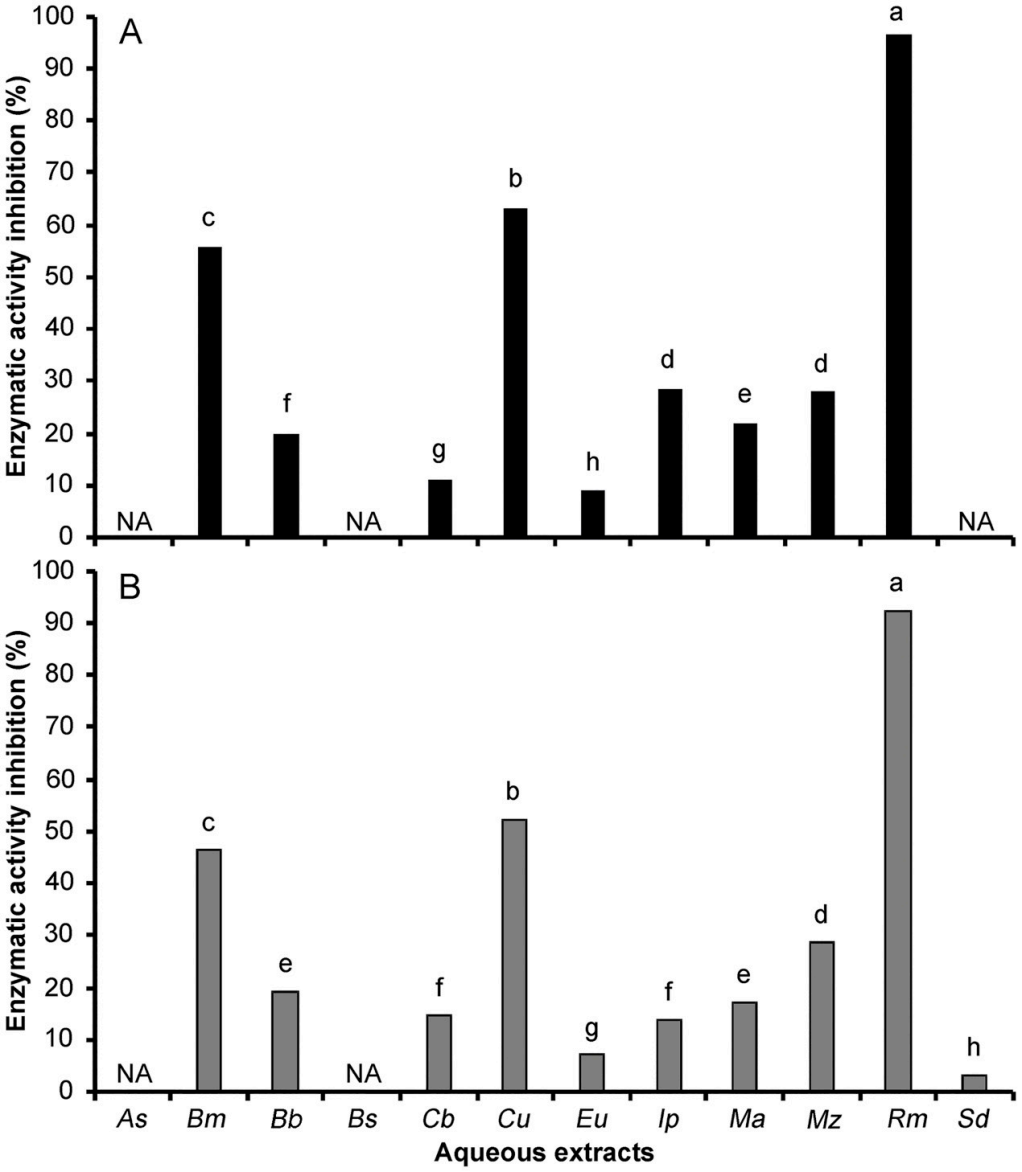
Inhibitory effect of aqueous extracts from Mayan medicinal plants on α-amylase (A) and α-glucosidase (B) enzymes. As = *Anthurium schlechtendalii*, Bm = *Bonellia macrocarpa*, Bb = *Bravaisia berlandieriana*, Bs = *Bursera simaruba*, Cb = *Capraria biflora*, Cu = *Coccoloba uvifera*, Eu = *Echites umbellata*, Ip = *Ipomoea pes-caprae*, Ma = *Malvaviscus arboreus*, Mz = *Manilkara zapota*, Rm = *Rhizophora mangle*, Sd = *Solanum donianum*, NA = Not active, a-h = different letters in the same enzymatic inhibition assay indicate statistical difference p < 0.05.

The anti-hyperglycemic activity of the aqueous extracts was investigated on the α-amylase and α-glucosidase enzymes, and inhibition percentage was calculated. As control, ascarbose showed inhibition of 50% at 29.59 μg/mL. Among the plants studied, the α-amylase inhibition percentage ranged from 8.90 to 96.74 (Fig 3A) and the α-glucosidase inhibition percentage ranged from 3.02 to 92.03 (Fig 3B). *R*. *mangle* was the species that registered the highest biological activity (96.74% and 92.03%), which could fortify the ethnomedicinal knowledge reported by the interviewees, although we tested the *R*. *mangle* leaves. Andrade-Cetto et al. [41] reported 17% α-glucosidase inhibition at 1mg/mL of *R*. *mangle* bark, and in this study the α-glucosidase inhibition of *R*. *mangle* leaves was 5.6-folds more active. The aqueous extract of *R*. *mangle* leaves can function as α-amylase and α-glucosidase inhibitor and might possess beneficial properties against diabetes mellitus. These activities may be a result of their content of phenols and flavonoids. Inhibitors of α-amylase and α-glucosidase delay the breakdown of carbohydrate in the small intestine and decrease the postprandial blood glucose excursion levels in diabetic patients [42]. In this regard, Fariba et al. [43] studied α-amylase inhibitors identified from medicinal herbs and highlighted that a number of inhibitors belonging to the flavonoid class are capable of inhibiting α-amylase activities.

### Antimicrobial activity

#### Human antimicrobial activity

**Fig 4:**
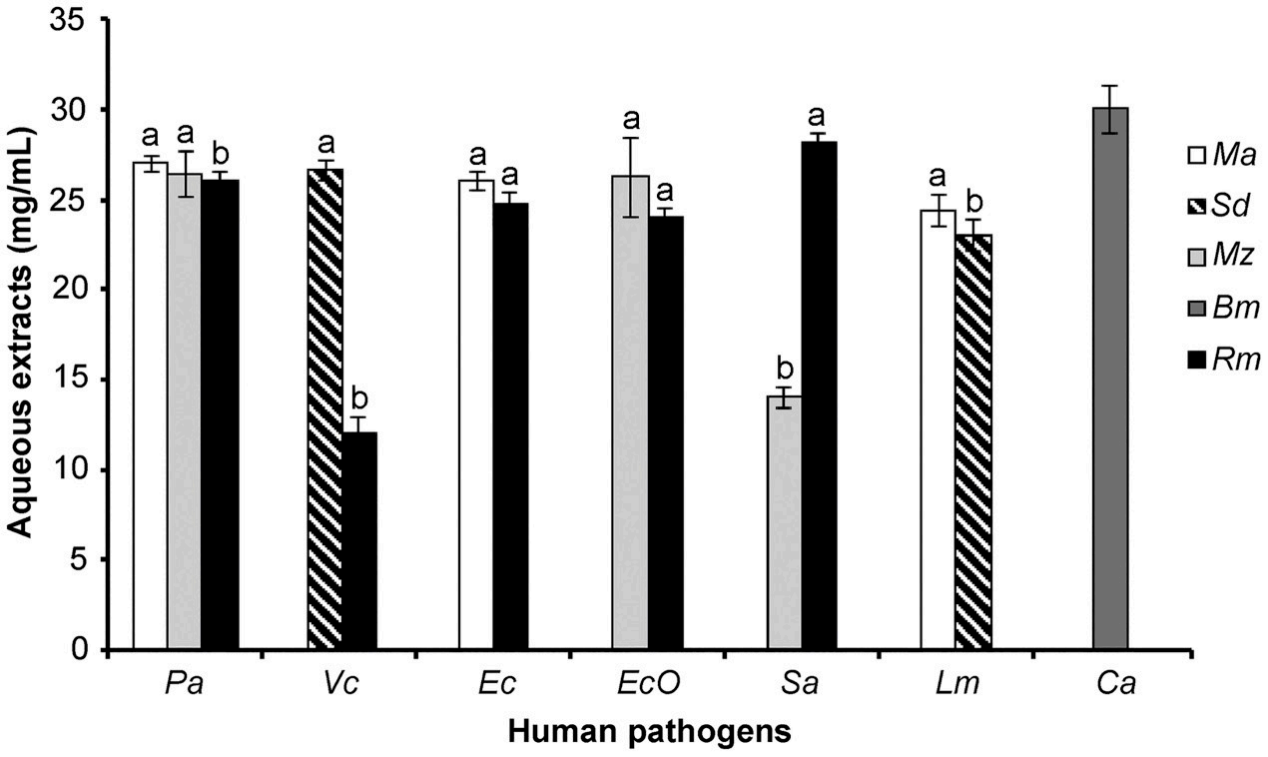
Aqueous extracts from Mayan medicinal plants evaluated against human pathogens. *Malvaviscus arboreus* (Ma), *Solanum donianum* (Sd), *Manilkara zapota* (Mz), *Bonellia macrocarpa* (Bm), *Rhizophora mangle* (Rm). Pa = *Pseudomonas aeruginosa*, Vb = *Vibrio cholerae*, Ec = *Escherichia coli*, EcO = *Escherichia coli* O157:H7, Sa = *Staphylococcus aureus*, Lm = *Listeria monocytogenes*, Ca = *Candida albicans*, a-b = different letters in the bars indicate statistical difference (p < 0.05).

Five of the twelve evaluated aqueous extracts presented antibacterial activity against different microorganisms (Fig 4), showing well-defined inhibition halos. *Rhizophora mangle* extract inhibited both Gram-positive and Gram-negative bacteria. It was effective against *V*. *cholera*, *E*. *coli* and *E*. *coli* 0157:H7 strains related to gastrointestinal diseases, with this being the first report as an antibacterial agent against *V*. *cholera* and *E*. *coli* 0157:H7. Also, ethanol extracts of *R*. *mangle* leaves, bark and roots showed moderate activity against *E*. *coli* and *Salmonella typhi* (1 mg/mL) [32]. Further, *R*. *mangle* inhibited opportunistic pathogens, such as *P*. *aeruginosa* and *S*. *aureus*. Montes de Oca et al. [44] studied the antimicrobial potential of *R*. *mangle* extract against *S*. *aureus* and *S*. *agalactia*, reporting minimum inhibitory concentrations of 8 mg/mL, in comparison with 28.2 mg/mL obtained in our study; the difference could be attributed to the part of *R*. *mangle* used by these authors, which was not reported, and also to the geographical origin of the plant. In our study, *M*. *zapota* aqueous extract also inhibited both Gram-positive and Gram-negative bacteria, meaning that it was effective against *E*. *coli* O157:H7, *S*. *aureus*, and *P*. *aeruginosa*. This is the first report on bactericidal activity of *M*. *zapota* against the enterohemorrhagic strain *E*. *coli* O157:H7. In previous studies, acetone and aqueous extracts of *M*. *zapota* showed to be active against other human pathogens [34]. Also, ethyl acetate extract of *M*. *zapota* leaves and bark have been subjected to screening for antibacterial and antifungal properties [45]. Nair and Chanda [46] reported antimicrobial activity from methanol extract of *M*. *zapota* against different pathogenic strains, including *S*. *aureus*, *P*. *aeruginosa*, and *E*. *coli*, and aqueous extracts against only *S*. *aureus*. *Malvaviscus arboreus* extract proved to be an agent with antimicrobial potential against related foodborne illnesses such as *E*. *coli* 25922 and *L*. *monocytogenes*, inhibiting the growth of both strains. These data are consistent with those of Yasunaka et al. [47] who reported *M*. *arboreus* extract to be a bactericide against *E*. *coli* and *S*. *aureus* at concentrations > 1 mg/mL and 256 μg/mL, respectively. In contrast, in our study, *E*. *coli* was inhibited with 26 mg/mL, while *S*. *aureus* was not inhibited. It is worth mentioning that there are no reports on *M*. *arboreus* inhibiting the growth of *L*. *monocytogenes*, with this being the first report on this species as an antimicrobial agent against this pathogen.

*Solanum donianum* extract showed activity against *V*. *cholerae* and *L*. *monocytogenes*, with the latter being more susceptible to this plant extract, requiring 23 mg/mL. No reports on the antibacterial activities of this wild species were found, meaning that this is the first report on its biotechnological potential. Leaf organic extracts of another species of the same genus, S*olanum torvum*, were active (2.6 to 30 mm) against *Escherichia coli*, *Bacillus subtilis*, *Staphylococcus aureus*, *Salmonella species*, and *Pseudomonas aeruginosa* [48].

*Bonellia macrocarpa* extract did not show antibacterial activity; however, it was the only extract with antifungal activity, inhibiting *C*. *albicans*. The antifungal activity of an organic extract of *Bonellia albiflora* has been reported by Vera-Ku et al. [49] where concentrations higher than 1 μg/mL were used, inhibiting 50–55% of the growth of *C*. *albicans*. In the present study, it was found that a concentration of 30 mg/mL of aqueous extract of *B*. *macrocarpa* inhibited 100% of the pathogen, with MIC = 12.5 mg/mL. This high concentration required to cause inhibition could be due to the low quantities of active compounds present in the aqueous extract, which is made up of a cocktail of many compounds, such as peptides, carbohydrates, and secondary metabolites.

#### Plant antifungal activity

Most of the plants have never been tested *in vitro* against agronomically important crop pests, such as phytopathogenic fungi. In this study, only four plant extracts caused mycelium growth inhibition to two fungi.

**Fig 5:**
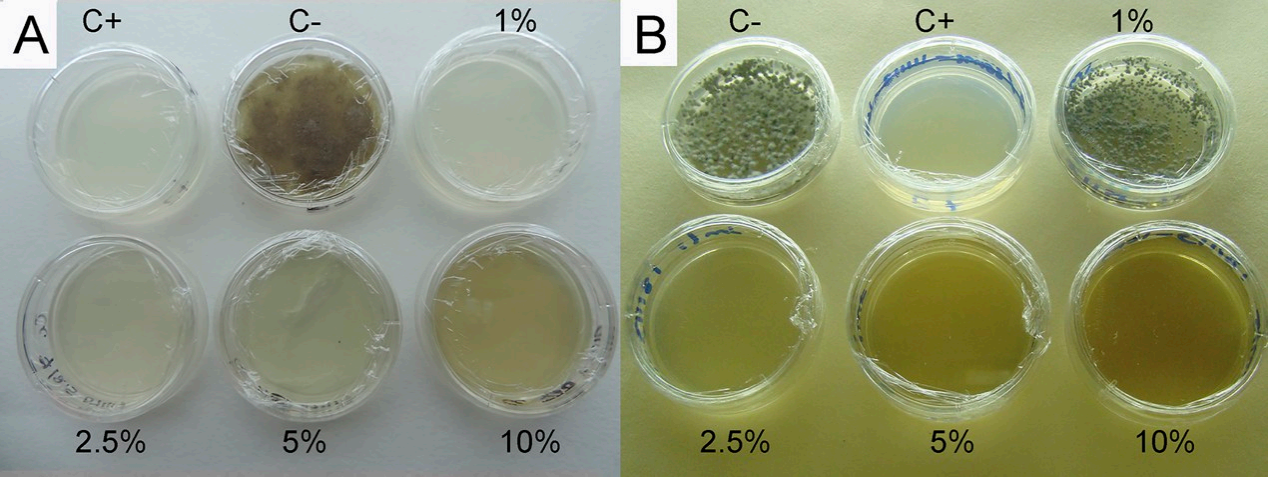
Antifungal activity of aqueous extracts from *Solanum donianum* vs *Colletotrichum capsici* (A), and *Bonellia macrocarpa* vs *Mycosphaerella fijiensis* (B) at 7 days postinoculation; negative control, PDB medium (C−); positive control, miconazole nitrate, 20 mg/mL (C+).

It has been reported that *Solanum* species (leaves, stem, roots, fruits, and hole plant) are the most potent against pathogenic microorganisms [50]. Antibacterial and antifungal activities have been reported from medicinal plants of this genus: *S*. *erianthum* [51] and *S*. *torvum* [52]. Nevertheless, there are no reports on *S*. *donianum* with antifungal activity against phytopathogenic fungi, so here we reported the inhibition of mycelia growth of *Colletotricum capsici* at 1% (w/v) (Fig 5A).

Aqueous extract of *M*. *zapota* at 50% (v/v) was tested against *C*. *capsici* and only 56% mycelium inhibition could be reached [53]. In the present study, *M*. *zapota* at 10% (w/v) showed 100% growth inhibition of *C*. *capsici* mycelium. No reports on *M*. *zapota* aqueous extracts against *M*. *fijiensis* have been published, and here we report a 100% mycelium growth inhibition at 5% (w/v) (S2 Fig).

This is the first report on *B*. *macrocarpa* with antifungal activity against *M*. *fijiensis*. The MIC was 2.5% with 100% mycelium growth inhibition (Fig 5B). García-Sosa et al. [54] reported that the organic extract of *Jacquinia flammea* roots (i.e. *Bonellia flammea*) showed antifungal activity against *C*. *gloeosporioides*. In our study the AE of *B*. *macrocarpa* was not active against *C*. *gloeosporioides*, being this difference due perhaps to the fact they are not the same species, and also because extraction method and different plant parts were used.

Finally, *R*. *mangle* aqueous extract has exhibited antimicrobial activity against *S*. *aureus* [55], but this is the first time it was tested against the phytopathogen *M*. *fijiensis*; in this study, the MIC was 10% with 100% mycelium growth inhibition. From the twelve medicinal plants screened, only four have the potential to be used in controlling fungi diseases of economically important crops, such as those caused by *M*. *fijiensis* in banana and *C*. *capsici* in pepper plants. The losses caused by these phytopathogenic fungi occur mainly as a direct reduction in the quality and quantity of the harvested product, and due to the use of synthetic fungicides to control them for many years in the field it is common to find fungal isolates that are less sensitive or even resistant to these fungicides [13]. *Mycosphaerella fijiensis* is responsible for black streak disease in banana and plantains, and *C*. *capsici* has been identified as the causative agent of anthracnose in multiple hosts, such as peppers (*Capsicum annuum* L. and *Capsicum chinense* Jacq.), papaya (*Carica papaya* L.) and physic nut (*Jatropha curcas* L.). For this reason, and to eliminate or reduce the negative impact of synthetic fungicides on the environment, it is very important to find an alternative to control fungi using natural products. In our case, the botanical knowledge obtained from people of the villages of the coastal dune and mangrove of the Yucatan peninsula opens a window of opportunities to search for compounds with antifungal activity to control crop pathogens.

### Chromatographic profile

Searching a chemical backup of the twelve medicinal plants, the HPLC chromatographic profiles of the twelve aqueous extracts were obtained using a previously published technique [18]. The analysis of these chromatograms is based on the results shown in S2 Table. Several solvent systems were tested until peaks were well separated, with gradient mixtures of acidified water (pH 2.5 with TFA) and acetonitrile being the best to carry out this separation. All chromatograms were obtained using two UV wavelengths (λ = 254 and 350 nm) with a final running time set to *t*_R_ = 65 min (*t*_R_ = retention time). When comparing chromatograms at both wavelengths, we observed a major abundance of compounds at 254 nm in all the aqueous extracts. The chromatogram window was conveniently divided into three zones to classify peaks according to their polarity and retention time [high (0–15 min), medium (15–40 min), and low (40–65 min)]. The aqueous extract of *B*. *macrocarpa* showed a chromatogram (λ = 254 nm) with 37 very well resolved peaks; they were concentrated mainly in the medium polar region, which corresponds to 62.79% of total area (mAU*s). Peaks with a high concentration (35.94% of total area) were observed in particular at *t*_R_ = 26.373, 28.493, 30.200, and 31.667 min. This species only showed activity against *C*. *albicans*.

In the chromatogram of *M*. *arboreus*, a total of 68 peaks (λ = 254 nm) were observed. They were concentrated in the medium polar region (63.16% of total area); in particular, peaks at *t*_R_ = 20.120, 21.787, 23.287, 23.747, 26.780, 28.493, and 30.760 min appeared very concentrated, which correspond to 49.77% of total area (mAU*s). This species only showed good activity against *E*. *coli*. The chromatographic profile of aqueous extract of *M*. *zapota* showed 76 peaks under short UV wavelength (254 nm); these were mainly observed in the medium polar region (61.89% of total area). Three of them (*t*_R_ = 19.320, 23.200, and 30.580 min) appeared greatly concentrated (45.96% of total area); also two peaks were of high polarity (*t*_R_ = 2.927, 7.033 min; 22% of total area). This plant showed antioxidant, antihypertensive, and antimicrobial activities (against crop and human pathogens). The chromatogram of *R*. *mangle* showed a total of 36 peaks (λ = 254 nm); most peaks were located in the medium polarity region, which corresponds to 74.81% of total area (mAU*s). In particular, three of them appeared very concentrated (*t*_R_ = 14.073, 19.547, and 30.507 min; 65.84% of total area). This plant showed antimicrobial activity against *M*. *fijiensis*, *P*. *aeruginosa*, *E*. *coli*, and *S*. *aureus*, and great antioxidant and antihypertensive activities. The chromatogram of *S*. *donianum* showed 56 peaks (λ = 254 nm), most of them detected in the high polar region (66.09% of total area). Seven of them appeared well concentrated and were detected at *t*_R_ = 2.38, 2.46, 2.627, 2.973, 3.927, 4.08, and 12.327 min, which correspond to 50.13% of the total area. This plant showed great antihypertensive activity and moderate antimicrobial activity against *V*. *cholerae* and *L*. *monocytogenes*.

It is not frequent to find in the literature reports on chromatographic profiles using HPLC of aqueous extracts of medicinal plants with any biological activity. At least, not any profile is reported on the twelve aqueous extracts here studied, and we consider this analysis necessary to have a chromatographic backup of the samples with biological activity that will allow us, in the future, to compare with other active samples of the same species collected in different years or seasons.

In conclusion, 42% of the aqueous extracts of the wild and native species of the coastal dune and mangrove of Yucatan State, selected for their use in Mayan traditional medicine, have shown biological activities: *Rhizophora mangle* and *Manilkara zapota* as antioxidants; *Solanum donianum* as ACE inhibitor; *R*. *mangle* as α-amylase and α-glucosidase inhibitor; *Malvaviscus arboreus*, *S*. *donianum*, *M*. *zapota*, *Bonellia macrocarpa*, and *R*. *mangle* as human antimicrobials; and *S*. *donianum*, *M*. *zapota*, *B*. *macrocarpa*, and *R*. *mangle* as plant antifungals.

These results showed the biotechnological potential value of native, wild, and medicinal plant species. Also, they are significant enough to make them known to the society, which, we believe, will reduce the negative impact that exists on these species that are under the risk of disappearing, because they are not considered useful to our society, regardless of its ecological value, also unknown or unimportant nowadays.

These species might have several applications as food and pharmaceutical products. For this, additional studies should be carried out to isolate and identify the compounds involved in the biological activities of the aqueous extracts.

## Supporting information

S1 Appendix Approval of the research ethics committee. (PDF)

S1 Fig Geographic coordinates of the collection sites of the twelve native plants of the Yucatan coast. (TIF)

S2 Fig Antifungal activity of the aqueous extracts from *Manilkara zapota* vs *Mycosphaerella fijiensis* at 7 days postinoculation; negative control, PDB medium (C−); positive control, miconazole nitrate, 20 mg/mL (C+). (TIF)

S1 Table Phytopathogenic fungi isolates used in this study. (DOCX)

S2 Table HPLC chromatographic profiles of 12 aqueous extracts of plant species collected in the coastal region of the Yucatan peninsula. (DOCX)
