# Supplementary material for: Antioxidant, antihypertensive, anti-hyperglycemic, and antimicrobial activity of aqueous extracts from twelve native plants of the Yucatan coast
Source: PLoS One. 2019 Mar 27;14(3):e0213493. doi: 10.1371/journal.pone.0213493 (PMC6436768; doi:10.1371/journal.pone.0213493)
Supplement: S1 Appendix — (PDF) [file pone.0213493.s001.pdf]

UADY
CENTRO DE INVESTIGACIONES REGIONALES "DR. HIDEYO NOGUCHI"

COMITÉ DE ÉTICA EN INVESTIGACIÓN

Mérida, Yucatán, México, a 20 de enero de 2018

DICTAMEN			
1ª REVISIÓN	**FECHA:** 25 ENERO 2018	**CIRB-03-2018**	**PROTOCOLO**: INVESTIGACIÓN

**TÍTULO**	Las plantas de la duna costera y del manglar de la península de Yucatán son fuente potencial de metabolitos contra hongos patógenos de cultivo de importancia económica
RESPONSABLES: **Adscripción**	Dra. Cecilia Mónica Rodríguez García.
**DONDE SE REALIZARÁ EL ESTUDIO:**	Centro de Investigación Científica de Yucatán (CICY)
**Teléfonos:**	9992557711 **Correo-e:** koyi@cicy.mx

Dudas y observaciones acerca del protocolo

1. Este proyecto fue evaluado de forma colegiada y con apego a las normas y leyes vigentes internacionales y de nuestro país.
2. El riesgo de los sujetos de investigación es mínima (encuesta a los sujetos de investigación) Artículo 23, Reglamento de la Ley General de Salud en Materia de Investigación para la Salud.
3. Daño mínimo, no maleficencia, con justicia retributiva, confidencialidad y conservación en privado de la identidad del sujeto de estudio.

RECOMENDACIONES

Informe al Comité de Ética en Investigación cada año de los avances.

Entregar informe de proyecto finalizado

DICTAMEN: **APROBADO**

Atentamente

Fernando Isaías Puerto Manzano
VOCAL PRESIDENTE

Marco Antonio Torres Castro
VOCAL SECRETARIO
