# Supplementary figures and images for: Antioxidant, antihypertensive, anti-hyperglycemic, and antimicrobial activity of aqueous extracts from twelve native plants of the Yucatan coast
Source: PLoS One. 2019 Mar 27;14(3):e0213493. doi: 10.1371/journal.pone.0213493 (PMC6436768; doi:10.1371/journal.pone.0213493)

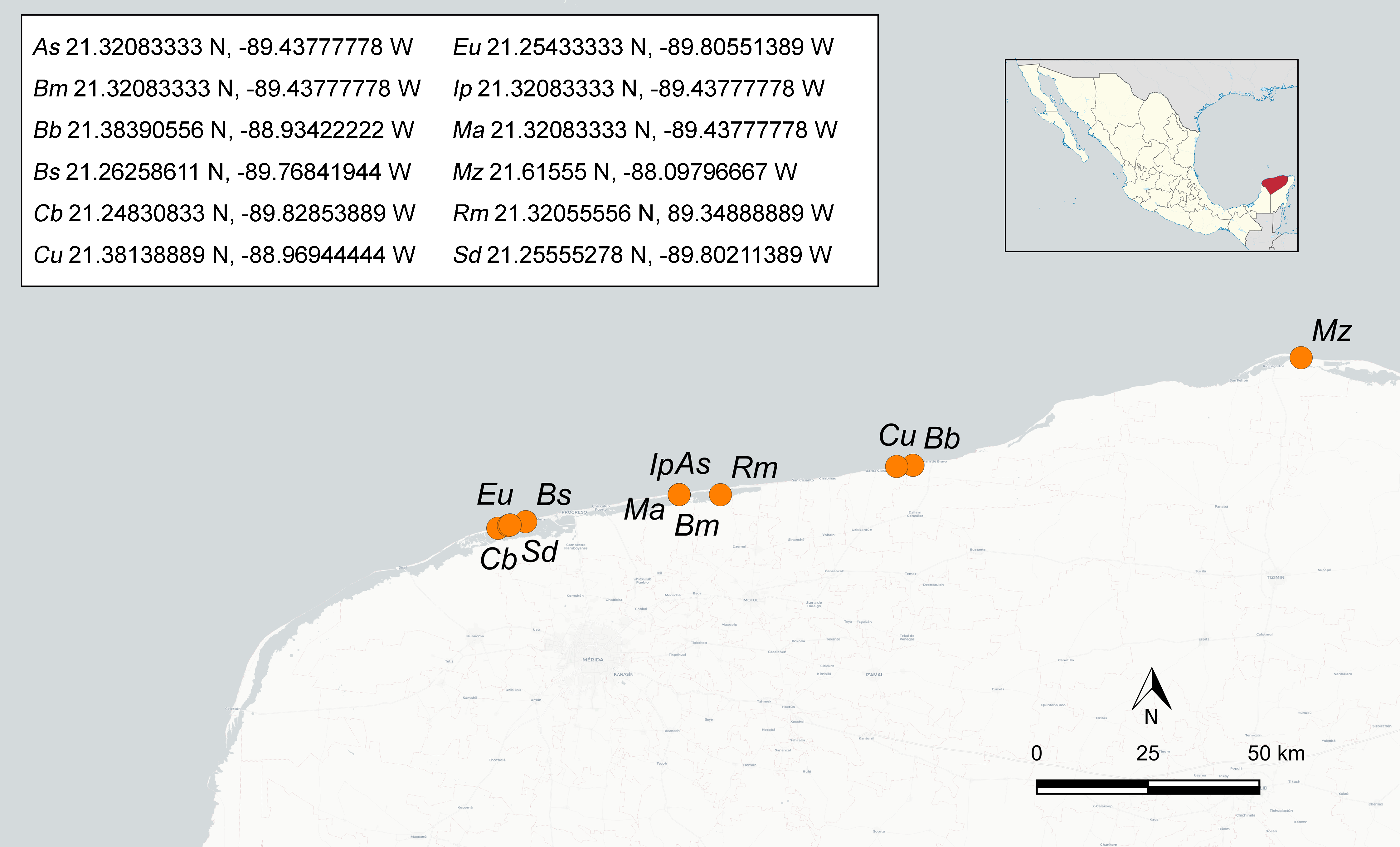

Supplement: S1 Fig — (TIF) [file pone.0213493.s002.tif]

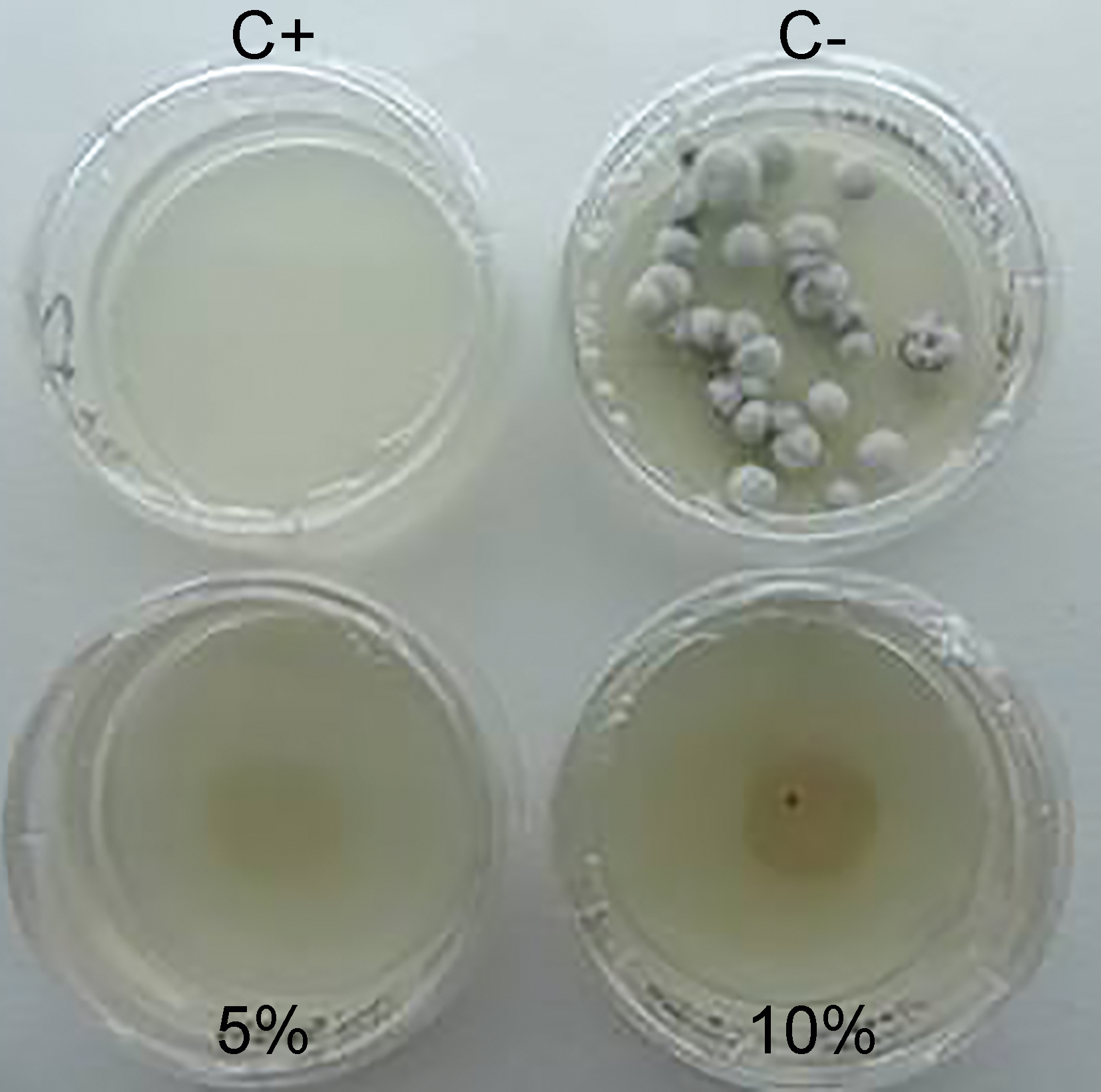

Supplement: S2 Fig — (TIF) [file pone.0213493.s003.tif]
