# Supplementary material for: Antioxidant, antihypertensive, anti-hyperglycemic, and antimicrobial activity of aqueous extracts from twelve native plants of the Yucatan coast
Source: PLoS One. 2019 Mar 27;14(3):e0213493. doi: 10.1371/journal.pone.0213493 (PMC6436768; doi:10.1371/journal.pone.0213493)
Supplement: S1 Table — (DOCX) [file pone.0213493.s004.docx]

**S1 Table. Phytopathogenic fungi isolates used in this study.**

| **Isolate** | **Species** | **GenBank accession #** | **Origin** |
| --- | --- | --- | --- |
| VF11 | *Fusarium oxysporum* | KR869782 | *Vanilla planifolia* |
| CGP6 | *Colletotrichum gloeosporioides* | JX982231 | *Carica papaya* |
| Ccg7 | *Colletotrichum capsici* | HM450129 | *Carica papaya* |
| JG52 | *Alternaria alternata* | KJ412493 | *Jatropha curcas* |
| VF9 | *Fusarium verticillioides* | KR869783 | *Vanilla planifolia* |
