# Supplementary material for: Antioxidant, antihypertensive, anti-hyperglycemic, and antimicrobial activity of aqueous extracts from twelve native plants of the Yucatan coast
Source: PLoS One. 2019 Mar 27;14(3):e0213493. doi: 10.1371/journal.pone.0213493 (PMC6436768; doi:10.1371/journal.pone.0213493)
Supplement: S2 Table — (DOCX) [file pone.0213493.s005.docx]

|  | **Key** | **Peaks^a^** | **mAU*s^b^** |  | | **Polarity^cd^** | |  | |
| --- | --- | --- | --- | --- | --- | --- | --- | --- | --- |
| **Species** |  |  |  | **High** | | **Medium** | | **Low** | |
|  |  |  |  | **(#, %)** | | **(#, %)** | | **(#,%)** | |
| *Anthurium schlechtendalii* | As | 56 | 2.345E+09 | 19 | 63.27 | 30 | 34.88 | 7 | 1.85 |
| *Bonellia macrocarpa* | Bm | 37 | 7.054E+09 | 12 | 29.57 | 20 | 62.79 | 5 | 7.64 |
| *Bravaisia berlandieriana* | Bb | 46 | 5.181E+09 | 25 | 79.6 | 17 | 20.11 | 4 | 0.29 |
| *Bursera simaruba* | Bs | 61 | 4.094E+09 | 30 | 56.91 | 22 | 36.85 | 9 | 6.24 |
| *Capraria biflora* | Cb | 21 | 9.728E+09 | 16 | 92.1 | 3 | 5.84 | 2 | 2.06 |
| *Coccoloba uvifera* | Cu | 96 | 3.756E+09 | 34 | 20.86 | 58 | 79.06 | 4 | 0.08 |
| *Echites umbellatus* | Eu | 56 | 2.048E+09 | 20 | 55.1 | 35 | 44.72 | 1 | 0.18 |
| *Ipomoea pes-caprae* | Ip | 45 | 3.952E+09 | 17 | 49.46 | 20 | 15.11 | 8 | 35.43 |
| *Malvaviscus arboreus* | Ma | 68 | 1.821E+09 | 24 | 36.5 | 26 | 63.16 | 3 | 0.34 |
| *Manilkara zapota* | Mz | 76 | 3.364E+09 | 34 | 38.11 | 42 | 61.89 | 0 | 0 |
| *Rhizophora mangle* | Rm | 36 | 5.049E+09 | 14 | 22.95 | 19 | 74.81 | 3 | 2.24 |
| *Solanum donianum* | Sd | 56 | 3.295E+09 | 32 | 66.09 | 22 | 30.15 | 2 | 3.76 |
|  |  |  |  |  |  |  |  |  |  |

**S2 Table. HPLC chromatographic profiles of 12 aqueous extracts of plant species collected in the coastal region of the Yucatan peninsula**

^a^Total number of peaks (254 nm)

^b^Total area

^c^ Number (#) of peaks by polarity

^d^Percentage (%) of area by polarity
